# Convergence of biofilm successional trajectories initiated during contrasting seasons

**DOI:** 10.3389/fmicb.2022.991816

**Published:** 2022-09-16

**Authors:** Jing Wang, Marc Peipoch, Xiaoxiao Guo, Jinjun Kan

**Affiliations:** ^1^Tianjin Key Laboratory of Animal and Plant Resistance, Tianjin Key Laboratory of Conservation and Utilization of Animal Diversity, Tianjin Normal University, Tianjin, China; ^2^Stroud Water Research Center, Avondale, PA, United States

**Keywords:** stream biofilm, community structure, succession, convergence, contrasting season

## Abstract

Biofilm communities play a major role in explaining the temporal variation of biogeochemical conditions in freshwater ecosystems, and yet we know little about how these complex microbial communities change over time (aka succession), and from different initial conditions, in comparison to other stream communities. This has resulted in limited knowledge on how biofilm community structure and microbial colonization vary over relevant time scales to become mature biofilms capable of significant alteration of the freshwater environment in which they live. Here, we monitored successional trajectories of biofilm communities from summer and winter in a headwater stream and evaluated their structural state over time by DNA high-throughput sequencing. Significant differences in biofilm composition were observed when microbial colonization started in the summer vs. winter seasons, with higher percentage of algae (Bacillariophyta) and Bacteroidetes in winter-initiated samples but higher abundance of Proteobacteria (e.g., Rhizobiales, Rhodobacterales, Sphingomonadales, and Burkholderiales), Actinobacteria, and Chloroflexi in summer-initiated samples. Interestingly, results showed that despite seasonal effects on early biofilm succession, biofilm community structures converged after 70 days, suggesting the existence of a stable, mature community in the stream that is independent of the environmental conditions during biofilm colonization. Overall, our results show that algae are important in the early development of biofilm communities during winter, while heterotrophic bacteria play a more critical role during summer colonization and development of biofilms.

## Introduction

Ecological succession, as the process by which species composition of biological communities change over time in a moderately predictable manner, is one of many fundamental concepts in ecology that were originally conceived from the observation of macroorganisms and is now being integrated into microbial ecology (Prosser et al., 2007; Fierer et al., 2010; Chapman et al., 2013; Jiao et al., 2016). Since then, a great deal of research on microbial succession has focused on biofilms in stream ecosystems as model systems that aggregate multiple species of algae, bacteria, fungi and protozoa (Rickard et al., 2003). Because biofilms play a critical role in energy flow and nutrient processing in headwater streams and rivers (Battin et al., 2008), successional patterns in stream biofilms have direct implications for our general understanding of freshwater ecosystems and their contribution to global carbon dynamics (Marx et al., 2017; Watts et al., 2018).

To fully comprehend and adequately predict biofilm succession, it is first necessary to individually characterize its multiple stages: initial (colonization), primary (assembly and development), and late (maturation; Fierer et al., 2010). Previous studies have extensively linked biofilm community assembly and development to a myriad of physical (Lyautey et al., 2005; Nemergut et al., 2007) and biological controls (Besemer et al., 2007). For instance, water shear force (Rickard et al., 2004), photic conditions (Rao et al., 1997) or nutrient dynamics (Sekar et al., 2002) are among the most important factors shaping the composition and diversity of microbial biofilms over time (Rickard et al., 2004; Lyautey et al., 2005; Besemer et al., 2007). Additionally, turnover of founder species during biofilm succession is an important ecological process shaping community succession (Brislawn et al., 2019). Community assembly can be also controlled by stochastic processes of speciation and extinction, as well as variation in dispersal capabilities among species (Zhou et al., 2013; Dini-Andreote et al., 2015). While patterns and mechanisms of biofilm community assembly have received much attention, much less is known about the importance of initial conditions for biofilm development and/or the timescale at which biofilm communities can reach maturity or alike.

The type and availability of microbial species that colonize benthic substrates influence the following successional trajectory. Stevenson (1983) first suggested the role of diatoms as early-colonizing agents with the ability to alter the microscopic environment and provide new, three-dimensional substrates for later heterotrophic colonization. Distinct seral stages in stream biofilm succession have been related to green algae, diatoms and cyanobacteria respectively, with water velocity and light conditions determining their chronological order (Stevenson et al., 1991; Biggs et al., 1998; Gosselain et al., 1998; Elliot et al., 2000; Wellnitz and Brader, 2003). Indeed, early colonization of stream biofilms is predominantly driven by algae due to the architectural advantage of modulating the microenvironment for other microbes (Richard et al., 2017). However, others have found a major role for heterotrophic organisms (e.g., bacteria) in biofilm colonization when light is limiting and organic carbon supply is abundant (Martiny et al., 2003). For instance, heterotrophic bacteria dominated at the beginning of biofilm development and were progressively reduced by protozoan grazing pressure (Rao et al., 1997). Ultimately, both the dominant microbial species in the water column and concurrent environmental conditions will influence colonization success and early development stages of epilithic biofilms after disturbance. As biofilm communities develop over time, they are likely to become less sensitive to environmental influence and more driven by their own microscopic conditions and interactions on the local habitat.

The capacity of epilithic biofilm communities to reach maturity—a final state characterized by high biomass, material recycling, and complex structure—is likely limited by the high-frequency disturbance regime of stream ecosystems. However, previous studies have observed the convergence of early biofilm communities into a somewhat mature biofilm community (Besemer et al., 2007) and estimated maturity periods varied from 60 to 100 days (Jackson et al., 2001; Besemer et al., 2012). Others have characterized biofilm development and maturity using specific individual metrics such as biomass (Tank and Dodds, 2003), bacterial density (Porter and Feig, 1980), cell lysis (Bayles, 2007), or biofilm detachment rates (Morgenroth and Wilderer, 2000). But the existence of a singular biofilm maturity state, the accuracy of our current estimation for this maturity period, and the length of it compared to the regular timeframe in between stream disturbances remain poorly characterized.

Here, we evaluate epilithic biofilm colonization, development and maturity at multiple locations and seasons with contrasting conditions of water temperature and light availability to compare how environmental stress and initial successional stages influence the trajectory of biofilm succession and maturity. We do so by using periphytometers, arrays of glass slides held in polycarbonate recesses covered by a cage, that minimize biofilm exposure to flooding disturbance and grazing pressures, and thus emphasizing the role of the baseflow physical conditions (light and temperature) and trophic interactions on biofilm community succession. Time series samples were collected and biofilm community structures were characterized with 16S rRNA gene high-throughput sequencing. The results and mechanisms underlying the observations will reveal the dynamics of biofilm communities from different seasons, and further broaden our understanding in development of freshwater biofilms and their potential biogeochemical roles in lotic ecosystems.

## Materials and methods

### Experimental design and sample collection

**Figure 1:**
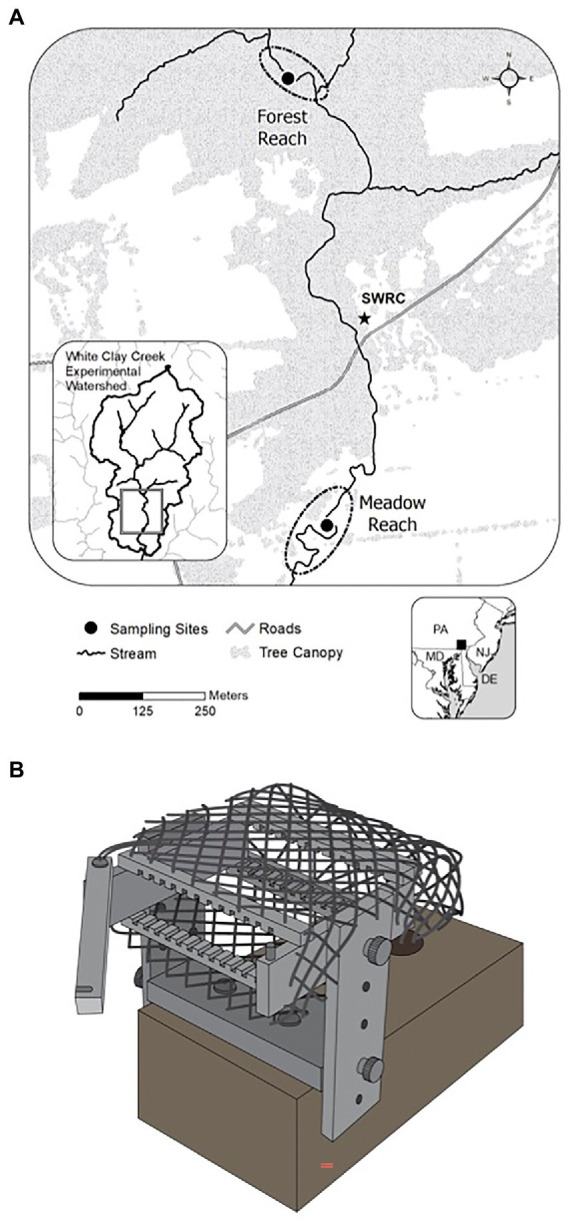
Map of the experimental locations at White Clay Creek **(A)** and a schematic design of a periphytometer **(B)**.

The study sites are located in White Clay Creek (WCC), a third-order watershed covering 277 km^2^ of the South-Eastern Pennsylvania Piedmont, Chester County, PA (39.858563°N, 75.783270°W, Figure 1A). The physical and chemical properties of surface water at WCC such as temperature, conductivity, and discharge have been monitored since 1968.^1^ Average baseflow in WCC is 86 L s^−1^, with a minimum of 66 L s^−1^ during the low-water period (August–October) and a maximum of 110 L s^−1^ through the high-water period between February and April. We selected two locations in the East Branch of WCC with contrasting riparian cover: one of them characterized by a typical meadow reach (39.858563°N, 75.783270°W) and the other located in a forest reach (39.86336°N, 75.78479°W; Figure 1A). A total of 24 glass slides were inserted into each periphytometer (Figure 1B), which was mounted on a heavy brick and stabilized underneath the water. Periphytometers (one at each site) were deployed first in the summer (3rd August 2011) and then again in the following winter (24th January 2012). All periphytometers (*N* = 4; summer meadow, summer forest, winter meadow, and winter forest) were in the water for a total of 12 months. At each sampling time, the biofilms growing on one side of the glass slide’s surface were collected by scraping all the biomass with the sterilized plastic scraper into sterilized Eppendorf tubes. The glass slide with the other side of biofilm were rinsed and preserved in 4% formaldehyde immediately for morphological observation. The slide was sampled the other day for the first month, biweekly for the second month, and then monthly till the end of the experiment. Due to the low biomass accumulated for the initial stage, only samples with successful amplifications were included in downstream analyses. In total, 15 summer and 14 winter succession samples were analyzed.

### DNA extraction, PCR and high-throughput sequencing

The genomic DNA from selected biofilm samples were extracted by using the Power Soil DNA Extraction kit (MoBio Laboratories, Carlsbad, CA, United States) following the manufacturer’s instruction. The concentration of DNA was determined with an ND-2000 Nanodrop spectrometer (Thermal Scientific, Wilmington, DE) by measuring the spectrophotometric absorption at 260 nm; the quality of DNA was accessed by the absorbance ratios at 260/280 nm and 260/230 nm, respectively.

In order to investigate the detailed community structure during the process of natural succession, partial 16S rRNA gene fragments (V1-V3) were used for 454 pyrosequencing. The forward primer was 27F (in bold), fused to the 454-titanium sequencing primer A adapters (5′-CGTATCGCCTCCCTCGCGCCATCAG**AGAGTTTGATYMTGGCTCAG**-3′), and the reverse primer was 534R primer, fused to variable key tags for multiplexing and to the 454 Titanium sequencing primer B (5′-CTATGCGCCTTGCCAGCCCGCTCAGxxxxxx**TYACCGCGGCTGCTGG** where the x region represents the various key tags and the primer sequences are in bold). PCR reactions (50 μl) consisted of primers (1.0 μl; 10 μM each), 1 μl template DNA (10–80 ng μl^−1^), and 45 μl 1.1× Platinum^®^ PCR SuperMix High Fidelity (Invitrogen, Carlsbad, CA), 0.5 μl 100× bovine serum albumin (BSA) and 1.5 μl RNase free water. Mixtures were denatured (95°C, 5 min), followed by 28 cycles of denaturation (95°C, 30 s), annealing (55°C, 30 s), and extension (68°C, 45 s) with a final extension (68°C, 7 min). The PCR amplicons were visualized on an agarose gel and purified using the Invitrogen ChargeSwitch PCR cleanup kit (Invitrogen, Carlsbad, CA).

The PCR amplicons were pooled in equimolar concentrations, and the purity, concentration and size were estimated using an Agilent 2100 Bioanalyzer (Agilent Technologies, Inc., Waldbronn, Germany) prior to performing emulsion reactions. Emulsion reactions of paired samples and sequencing were performed on a 454 Life Sciences Genome Sequencer FLX using Titanium chemistry (Roche Diagnostics, Indianapolis, IN) with the unidirectional amplicon library sequencing protocol with emPCR Kit II (Roche) at the Perelman School of Medicine of the University of Pennsylvania.

### Analyses of community structure

Raw 454 Titanium sequences were sorted and separated by tag sequences with Geneious Prime^2^ and then the sequences were cleaned and analyzed using the DADA2 R package (Version 13.8, Callahan, 2016). The low-quality sequences and chimeras were removed, and only high-quality data with at least 300 bp were kept for downstream analysis. Rarefaction curves were generated within the DADA2 script, and amplicon sequence variants (ASVs) were normalized based on 99% coverage with a cutoff at 8,000 sequences per sample. Four samples were omitted due to low sequencing coverage. Sequences obtained in this study were deposited to NCBI GenBank with the accession no. PRJNA484224.

A Naïve Bayes classifier^3^ was applied to assign the ASVs to taxa at 99% using the Silva classifier 132. Calculation of alpha and beta diversity of biofilm communities was done using the “diversity” function in the “vegan” package with R statistical software (version 3.6.1; Oksanen et al., 2019). Non-metric dimensional scaling (NMDS) was performed based on Bray-Curtis distance matrices in order to describe variations in biofilm compositions. Analysis of similarity and difference of community structure was performed by Bray-Curtis similarities and ANOSIM between summer/meadow and winter/forest successions. The NMDS, Bray–Curtis similarity and ANOSIM analyses were performed using “metaMDS,” “phyloseq” and “anosim” functions in the “vegan” package; phylogenetic distances based on Faith PD was calculated by using “picante” and “ape” packages in R (R version 4.2.1) and phylogenetic tree was constructed with MEGA 11.

## Results

### Environmental conditions and biofilm composition during summer and winter succession

Stream water temperatures were higher in summer than winter time (Table 1). Due to sun light and lacking of canopy cover, the water temperature at meadow site was 0.5°C higher than forest sites in summer but with no significances. Photosynthetically active radiation (PAR) was also higher in the summer and at the meadow compared to PAR values in the winter and forest sites (Table 1). Stream water chemistry (pH, DOC, NH_4_-N, NO_3_-N, PO_4_^3−^, SO_4_^2−^ etc.) were similar within each season (Table 1) and only showed marginal difference between sites (Table 1).

**Table 1:** Environmental measurements (monthly average) for forest and meadow reach in summer and winter.

Parameters	Forest		Meadow	
	Summer (August)	Winter (January)	Summer (August)	Winter (January)
Water Tm (°C)	19.85	4.53	20.36	4.22
Light PAR (mol Q photons m^−2^ day^−1^)	8.71	3.98	34.65	13.87
pH	7.28	7.47	6.75	7.46
Conductivity (μS/cm)	227.5	192.1	236.5	204
Mean DOC (mg/L)	1.34	0.80	1.58	0.78
NH_4_-N (mg/L)	0.01	0.01	0.03	0.01
NO_3_-N (mg/L)	3.70	4.13	3.68	3.68
PO_4_^3−^ (mg/L)	0.02	0.01	0.02	0.01
Total Phosphorus (mg/L)	0.03	0.02	0.03	0.01
SO_4_^2−^	15.69	16.45	18.05	15.44
K (mg/L)	2.06	1.78	2.06	1.83
Mg (mg/L)	9.03	9.51	10.11	10.25
Na (mg/L)	8.07	7.62	8.52	7.76

Data source: https://www.hydroshare.org/resource/c29f39c122c3411c97a3bfb933f156a2/

**Figure 2:**
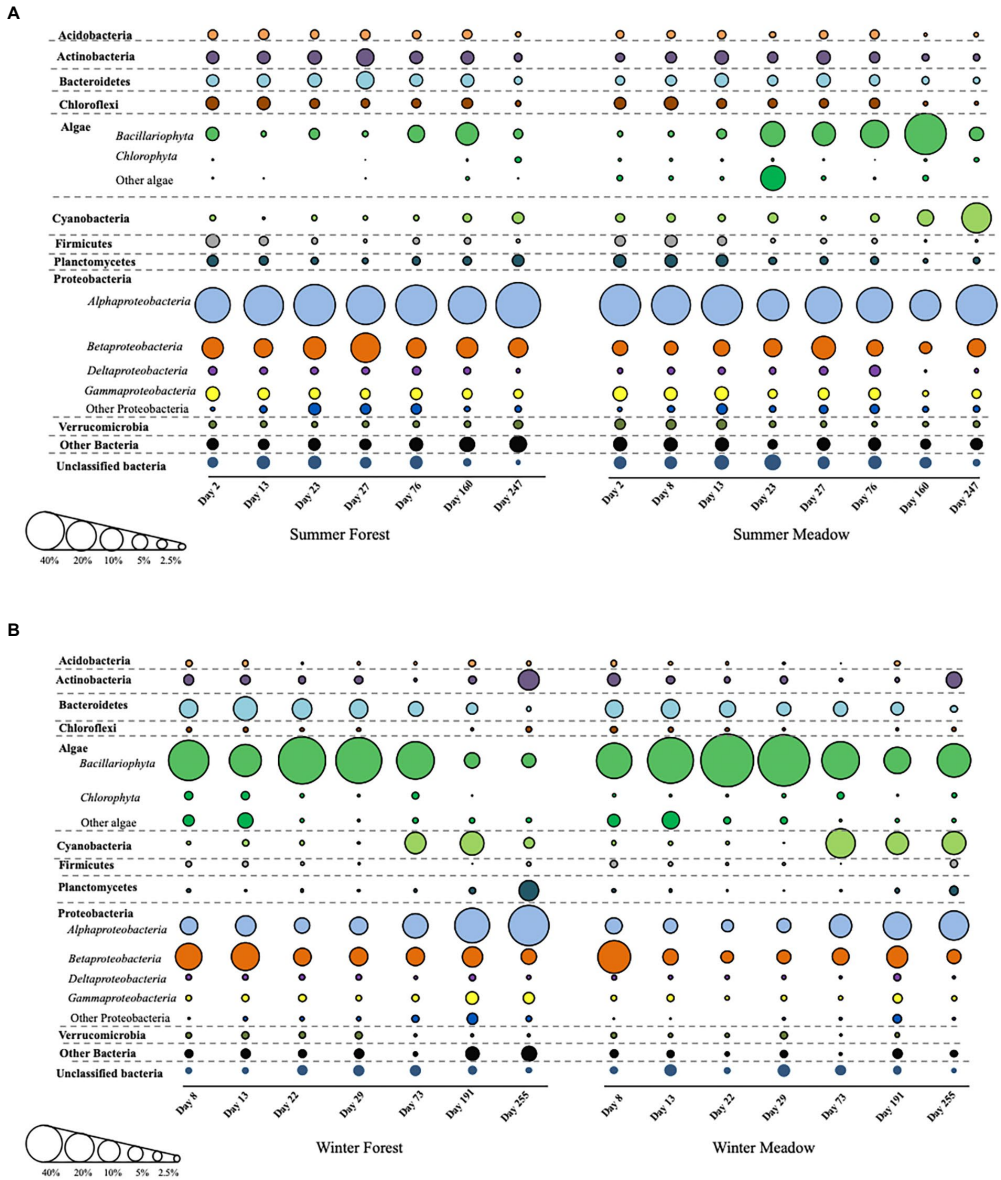
The detailed succession of periphyton community composition starting from summer **(A)** and winter **(B)**. Bubble sizes are corresponding to relative abundance of each group.

Biomass accumulation in all periphytometers was apparent in our series of photographs taken over the duration of the study (Supplementary Figure S1). Substantial variations in the composition and relative abundance of microbial taxa in the biofilm communities were observed at each site and between colonization season (Figure 2). Overall, successional patterns in biofilm community composition within each season were similar between meadow and forest sites, particularly for heterotrophic phyla (Figure 2). Major groups of heterotrophic bacteria identified in the biofilms were Acidobacteria, Actinobacteria, Bacteroidetes, Firmicutes, Planctomycetes, Proteobacteria and Verrucomicrobia (Figure 2). However, distinct compositional (at phylum level) changes occurred when comparing winter vs. summer colonization, especially at the early stages of biofilm development (Figure 2). In summer, algae and cyanobacteria steadily increased and became more abundant at later succession (Figure 2A). In addition, Proteobacteria maintained high abundance during the succession along with other groups including Actinobacteria, Bacteroidetes, and Planctomycetes. When biofilm colonization initiated in the winter, algae were more abundant than other heterotrophic phyla in the first 73 days, while cyanobacteria increased at the later succession (Figure 2B). Interestingly, Bacteroidetes showed a similar trend as algae, and decreased along with succession at both forest and meadow sites.

### Structural patterns of biofilm colonization, development, and maturity

**Figure 3:**
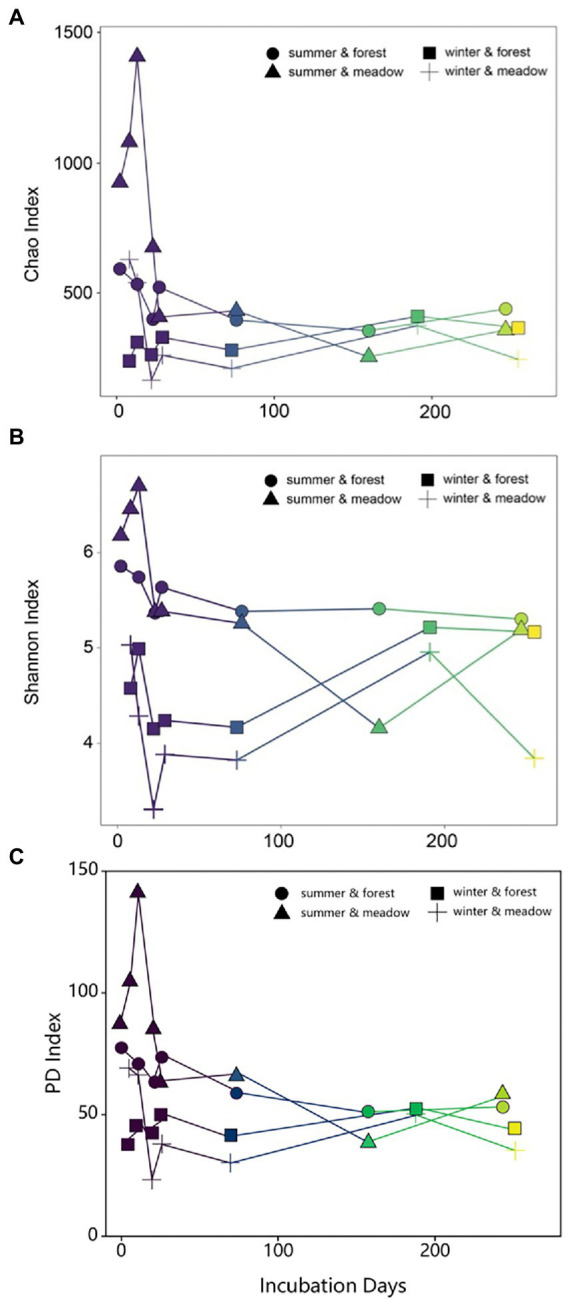
Changes of Chao **(A)**, Shannon **(B)** and PD **(C)** indices of periphyton communities during the succession.

Temporal patterns of biofilm diversity in summer-and winter-initiated biofilms showed similar trends over the study period, with dynamic changes from initial, primary to late development stages (Figure 3). Species richness (as determined by Chao index) decreased in the first 30 days and then remained fairly stable over the rest of the study period (Figure 3A). Similarly, the Shannon diversity index also dropped sharply at the early stage regardless of the site or season (Figure 3B). Later on biofilm development from summer showed relatively stable diversity over time, while winter colonization led to progressively increasing diversity until reaching similar diversity level as summer-initiated communities (Figure 3B). These results were further supported by similar biofilm community compositions across sites in the same season (mean Bray–Curtis similarity = 0.62, *p* = 0.198), but contrast with significant differences in community composition between colonization seasons (ANOSIM, *R* = 0.60, *p* = 0.001).

**Figure 4:**
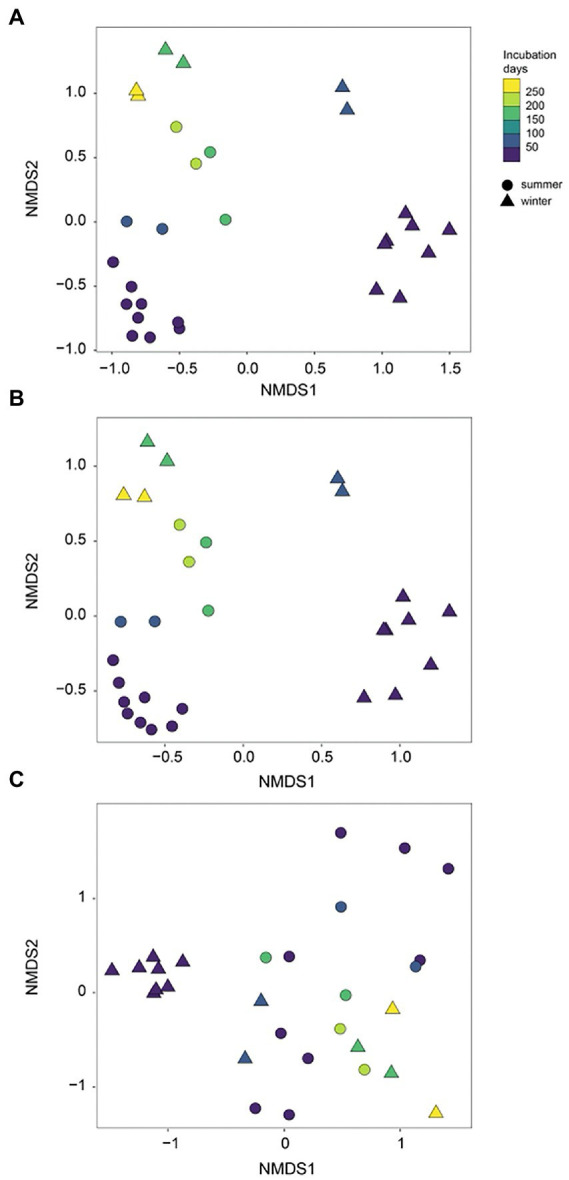
NMDS separation of periphyton communities in summer and winter succession: **(A)** all sequences; **(B)** heterotrophic bacteria only; **(C)** algae (chloroplast) + cyanobacteria only. Summer and winter succession are in different symbols and color gradients indicate the incubation time (days).

**Figure 5:**
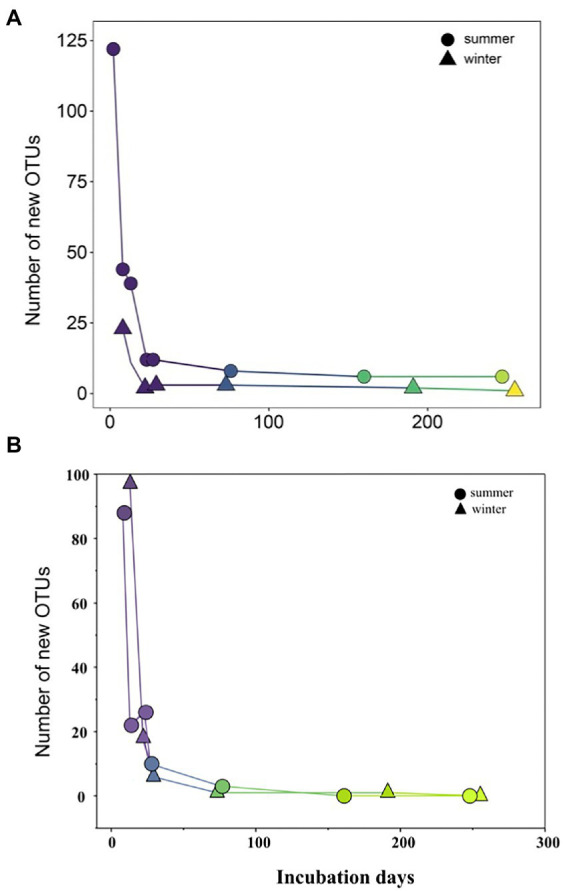
Newly occurred **(A)** and lost **(B)** microbial ASVs along summer and winter successions.

During initial and primary development stages, biofilm communities that developed over summer were distinctly separated from those that grew over winter (ANOSIM, *R* = 0.49, *p* = 0.001), indicating the impact of initial conditions for biofilm colonization (Figure 4A). Then biofilm community structure began to converge after 73 days of development showing high similarity from both seasons (Figure 4A). Similarly, the arrival of new taxa to biofilm communities over time decreased exponentially with almost identical exponents for both summer-and winter-colonized biofilms (*b* = −0.009 and −0.008, respectively), with <1 new taxa per week in biofilm communities after 73 days (Figure 5A). Simultaneously, loss of the existent taxa corresponding to the newly arrived taxa was also decreased sharply, with only one taxon lost after 73 days in winter and no more taxa was lost after day 160 in summer (Figure 5B). Separate NMDS analyses for heterotrophic bacteria corresponded well with the plot for the total community structure (Figure 4B), indicating a compositional dominance of heterotrophic bacteria during biofilm succession. In contrast, differences and separation of photosynthetic microbes (algae and cyanobacteria) between meadow and forest were shown at the early stages (Figure 4C). Eventually they converge after 200 days of incubation, but the temporal transition was not as distinct as the heterotrophic microbes (Figure 4B).

## Discussion

Depending on environmental conditions, mature biofilms can develop around 60–100 days (Jackson et al., 2001; Besemer et al., 2007, 2012). A large number of investigations have been conducted using microscopy or modeling calculation to reveal the structural variations in microbial biofilm developments (Acuña et al., 2004; Alpkvist et al., 2006; Schlafer and Meyer, 2017). With regard to detailed biofilm community structure during succession, by flume and tank experiment combined with high-throughput sequencing of 16S rRNA genes, Niederdorfer et al. (2016) revealed that the formation of stream biofilm was highly dependent on microbial lifestyle but not the planktonic communities from the surrounding environments. During the succession of stream biofilms, biofilm development has been confirmed to be dominated by slowly-but-efficiently growing microorganisms, as an alternative trade-off of dominancy over rate-yield strategy (Niederdorfer et al., 2017). However, these studies mainly focused on internal mechanisms of biofilm formation and surrounding influencing factors, but the seasonality effects and long-term stability of biofilm after maturation are poorly explored and investigated. In our study, the variations of each microbial group (genus/species level) from contrasting colonization seasons (winter vs. summer) were revealed by high-throughput sequencing, which could greatly improve our understanding of the attachment, assembly and maturation of biofilm initiated from different environmental condition.

Results from NMDS and diversity indices clearly showed three stages in succession process: initial (colonization), primary (assembly and development) and late (maturation; Besemer et al., 2007; Fierer et al., 2010). Due to the limited biomass of collection, our analysis likely missed the early initial colonization, especially in the cold season (Figure 3). However, as revealed by a previous study that turnover was the ecological process defining the biofilm community succession (Brislawn et al., 2019), our results also presented clear replacement of existing taxa with new species, illustrated by taxa loss and emergent of new taxa at each examined time point during succession, which was the outcome of an ecological process in stream biofilm succession. Dynamics of bacterial community during biofilm succession clearly defined three stages of ecological process. During the first few days, random dispersal of the suspending microbial sources from the water column is always in charge of the initial colonization for biofilm (Jackson, 2003; Battin et al., 2007), which could explain the increase of diversity in our study for the first several sampling time point. Hereafter, diverse microorganisms are enriched and assembled on the submerged surfaces *via* a stochastic process (Nelson et al., 2009; Lindström and Langenheder, 2012; Burns et al., 2016), known as ecological drift (Stegen et al., 2012), dispersal, and immigration (Chase and Myers, 2011) under nutrient or temperature fluctuation (Salcher et al., 2013; Dini-Andreote et al., 2015) and flow velocity (Besemer et al., 2007). At the second stage, the community of microbes continue growing with a demand of architecture establishment for biofilms as indicated by Veach et al. (2016). Since the successional trajectories of bacterial groups are different, biofilm community always results in species sorting process (Schmidt et al., 2014; Dini-Andreote et al., 2015), which is driven by selection, survival and growth ability of different species (Burns and Ryder, 2001). Finally, as the abundant groups outcompete rare species along with the development of biofilm (Jackson et al., 2001), quickly high competition between species occurred and was visualized by the sharp decrease of alpha and beta diversity indices for the first month (Figures 3A,B). Exclusion or replacement of closely related taxa at mature stage resulted in low PD value, suggesting shorter phylogenetic distances compared to initial stage (Figure 3C). This hypothesis was further supported by the phylogeny from early to late stage of the biofilm maturation, where the closely related taxa competed for similar niches and certain clades were lost or replaced by newly occurred species (Supplementary Figure S2). The decrease of diversity during developmental stage was also indicated in other successional study in freshwater biofilm (Besemer et al., 2007). Compared to primary stage, microbial diversity recovered and increased in the late stage (Figure 3), which is likely due to resource availability, niche development and habitat heterogeneity (Odum, 1969; Jackson, 2003; Ortiz-Álvarez et al., 2018). Nevertheless, biotic and abiotic conditions tend to drive the biofilm assembly mechanisms toward species sorting for later succession, where local environmental conditions are critical in determining the biofilm composition and distribution (Chase et al., 2009; Declerck et al., 2013; Ren et al., 2015). The re-establishment of highly competitive rather than taxonomic variable species might explain the decrease of diversity occurred at the last time point of our study (250 days; Figure 3; Margalef, 1963; Ortiz-Álvarez et al., 2018), which has not been extensively studied due to short incubation time in previous studies.

Different biofilm communities between winter and summer colonization were observed from this study, which reflected different adaption strategies for groups of microbes during the progress of succession. For initial colonization, the pioneer microbial groups with adhesion ability first appeared on the fairly bare substratum with a stochastic strategy (Veach et al., 2016). These pioneer species also exhibited high growth rate to favor a rapid development within the initially formed biofilms (Besemer et al., 2007). In our study, the composition of pioneer colonizers in summer were mainly heterotrophic bacteria such as Proteobacteria, Actinobacteria, and Chloroflexi etc., which are different from those starting in winter (e.g., algae and Bacteroidetes). Significant seasonal variations in bacterial succession in epilithic stream biofilms have demonstrated the temperature is a key factor in controlling the biofilm development and maturation (Lyautey et al., 2005; Lang et al., 2015). Meanwhile, certain microbial groups were found in both seasons and remained constantly abundant during the succession, such as Rhizobiales, Rhodobacteraceae and Sphingomonadaceae. These key microbial groups play vital roles in not only developing but also maintaining resilience to environmental disturbance through their fast growth rate, high degree of physiological flexibility or ability of evolution to environmental change (Allison and Martiny, 2008). Although distinct microbial communities were found in initial and primary stages for summer vs. winter, biofilm population structure converged in the late succession (Figures 3, 4). Such kind of succession has been described before as a random assembly of taxa first and then converted into similar community types due to local deterministic effects (del Moral, 1999). In a given habitat, the communities in later succession stages always contained higher similarity within the groups than in early succession stages (Besemer et al., 2007; Ortiz-Álvarez et al., 2018). This consistence of communities in successional process occurred regardless of the taxonomic composition, only with the exception of strong heterogeneous environmental gradients that might lead to historical contingency (Fukami, 2015).

In running streams, the selection pressure from the shear force favors the coaggregation partnership in multispecies biofilms more than the planktonic suspension in water (Rickard et al., 2003). For instance, predominant Cyanobacteria and Bacteroidetes occurred in streambed biofilms while the surface water contained high abundance of Actinobacteria (Zeglin, 2015). This further demonstrated the inconsistency between the microbial composition within biofilm and that from the surrounding bulk water, because the non-aggregating species would be washed away under flowing conditions. Shear rates (Rochex et al., 2008), violent disturbance and frequency (Burns and Ryder, 2001; Battin et al., 2007) all affect the composition and diversity patterns of epilithic biofilms. Further, nutrient availability was believed to impact the succession trajectories directly (Knelman et al., 2014; Fernández-Martínez et al., 2016). Based on the classification of nutrient utilization strategy, the Actinobacteria and Chloroflexi are oligotrophic-favored bacteria and they are negatively correlated with carbon mineralization rates (Fierer et al., 2007) or primary production (Li et al., 2019). In our results, a decreasing trend of these two groups of bacteria was observed with the growth of algae (Bacillariophyta and Chlorophyta) and cyanobacteria during the succession in both seasons, and similar response was observed for Firmicutes and Planctomycetes in summer. In contrast, Bacteroidetes prefer eutrophic conditions (Fierer et al., 2007) and they responded to algal growth in winter succession. In winter time, algae such as Bacillariophyta and Chlorophyta are the major component of biofilms (Battin et al., 2003; Arnon et al., 2007; Besemer et al., 2007; Artigas et al., 2012; Wilhelm et al., 2013), which is high in relative abundance for all winter succession sample, as well as the late stages of summer meadow succession. These algae serve as “ecosystem engineers,” provide substrates and architecture for connecting other heterotrophic bacteria in biofilm development (Rickard et al., 2002; Besemer et al., 2007). Moreover, algal exudates together with extracellular polymeric substances (EPS) played key roles for adhesion and stimulation of heterotrophic microorganisms to the substratum (Costerton et al., 1995; Carr et al., 2005; Samways et al., 2015), and/or provide major carbon source for heterotrophic microbes (Battin et al., 2001; Romaní et al., 2004), such as Bacteroidetes in this study. Thus, investigating the succession of entire biofilm communities provides great opportunities to investigate the complex associations between microorganisms, such as algae and heterotrophic microbiota.

## Conclusion

In summary, our findings reveal the detailed biofilm structure and their community trajectories during natural succession starting from different seasons in the White Clay Creek. Although distinct succession pattern from different seasons were observed in early stages, the biofilm community structure converged at later colonization stage (after over 70 days). Random dispersal and species sorting are likely responsible for initial, primary and late stages of biofilm succession. The convergence of biofilms succession suggests a stable, mature community in the stream that is independent of colonization seasons. Our study provides detailed characterizations of biofilm community structure and its succession from different seasons in a lotic freshwater ecosystem, expanding our current knowledge of ecological strategies with initial divergence but mature convergence in stream biofilm development.

## Data availability statement

The datasets presented in this study can be found in online repositories. The names of the repository/repositories and accession number(s) can be found in the article/Supplementary material.

## Author contributions

JW and JK: conceptualization. JK: experimental operation. JW and XG: writing – original draft preparation. JK and MP: writing – review and editing. JW, MP, and JK: funding acquisition. All authors contributed to the article and approved the submitted version.

## Funding

This study was supported by NSF LTREB program (DEB-1557063), Endowment Fund of Stroud Water Research Center, National Natural Science Foundation of China (52070143 and 41506182), Natural Science Foundation of Tianjin City (19JCZDJC40300), and China Scholarship Council.

## Conflict of interest

The authors declare that the research was conducted in the absence of any commercial or financial relationships that could be construed as a potential conflict of interest.

## Publisher’s note

All claims expressed in this article are solely those of the authors and do not necessarily represent those of their affiliated organizations, or those of the publisher, the editors and the reviewers. Any product that may be evaluated in this article, or claim that may be made by its manufacturer, is not guaranteed or endorsed by the publisher.

## Footnotes

^1^ https://www.hydroshare.org/resource/c29f39c122c3411c97a3bfb933f156a2/

^2^ https://www.geneious.com/

^3^ https://github.com/qiime2/q2-feature-classifier
