# Supplementary material for: Convergence of biofilm successional trajectories initiated during contrasting seasons
Source: Front Microbiol. 2022 Sep 16;13:991816. doi: 10.3389/fmicb.2022.991816 (PMC9522907; doi:10.3389/fmicb.2022.991816)
Supplement: Supplementary file 1 [file Data_Sheet_1.docx]

**Convergence of biofilm successional trajectories initiated during contrasting seasons**

**Jing Wang^1,2^, Marc Peipoch^2^, Xiaoxiao Guo^1^, and Jinjun Kan^2*^**

1Tianjin Key Laboratory of Animal and Plant Resistance, Tianjin Key Laboratory of Conservation and Utilization of Animal Diversity, Tianjin Normal University, Tianjin 300387, P.R. China

2Stroud Water Research Center, Avondale, PA 19311, USA

*Corresponding Author
[jkan@stroudcenter.org](mailto:jkan@stroudcenter.org)

**Funding**

This study was supported by NSF LTREB program (DEB-1557063), Endowment Fund of Stroud Water Research Center, National Natural Science Foundation of China (52070143, 41506182), Natural Science Foundation of Tianjin City (19JCZDJC40300), and China Scholarship Council.

**Acknowledgments**

We thank Laura Zgleszewski for her help in data analysis.

**Supplementary materials**

**Figure legends**

**Fig. S1** Slide photographs showing the succession of periphyton development. A, summer forest and summer meadow; B, winter forest and winter meadow.

**Fig. S2** Neighbor-joining phylogenetic tree showing the loss of closely related brunches (in colored brunches) or replacement by newly occurred taxa (in bold) from initial stage (A) to mature stage (B) during stream biofilm succession (Bootstrap values were 1,000 replicates, numbers in the brackets were the total value of the corresponding OUT at each succession stage).


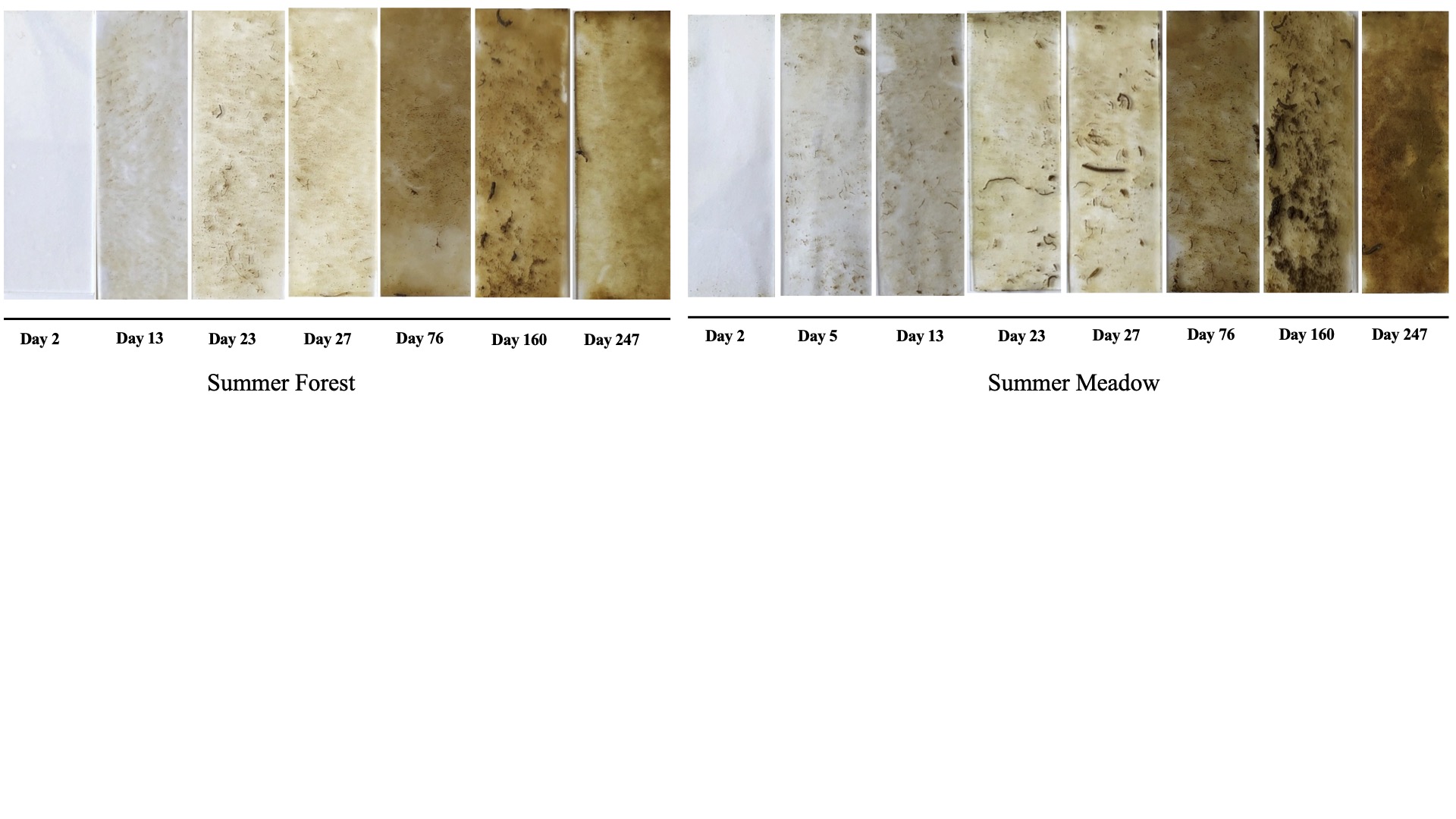


A


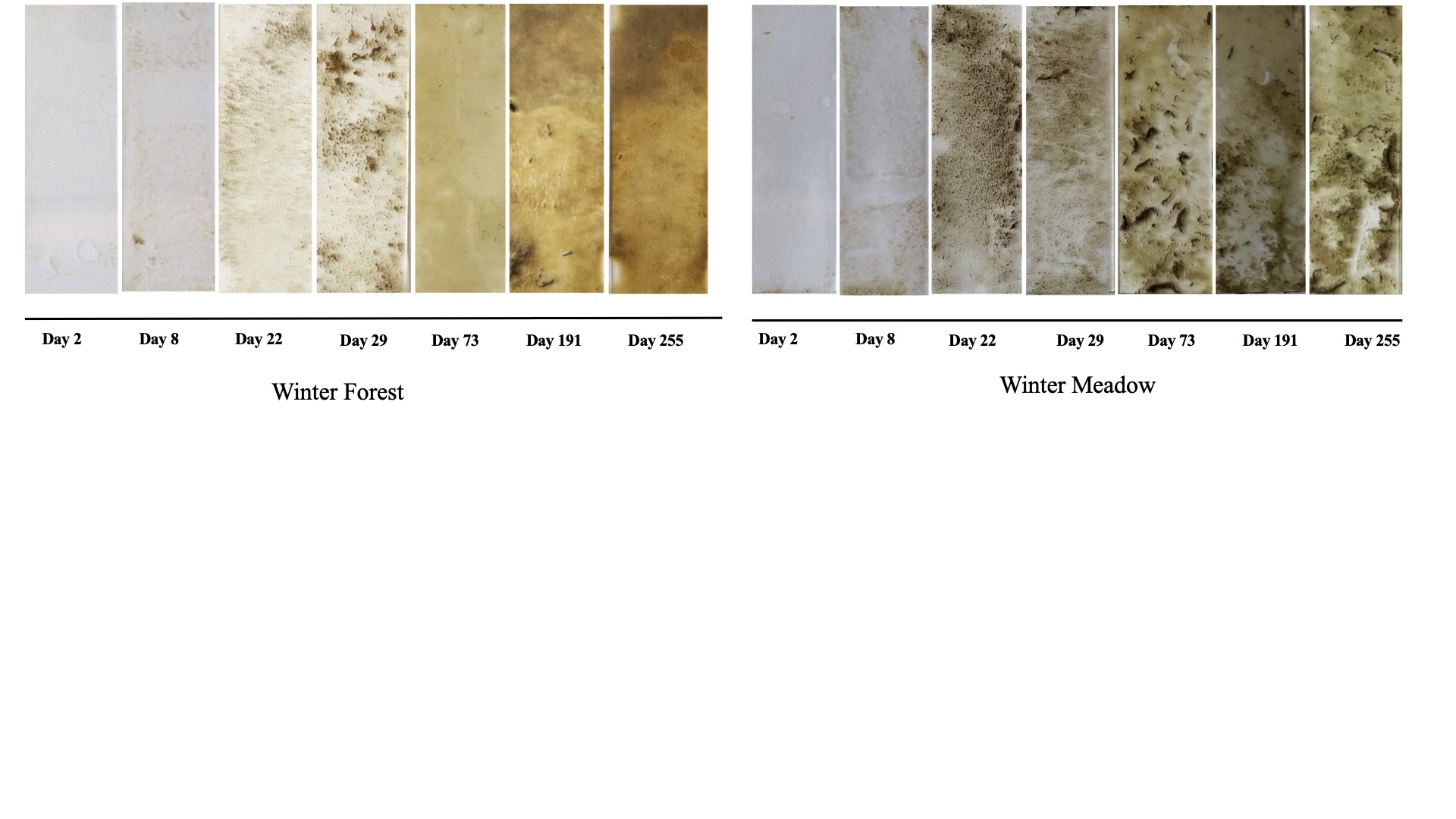


B

Fig. S1


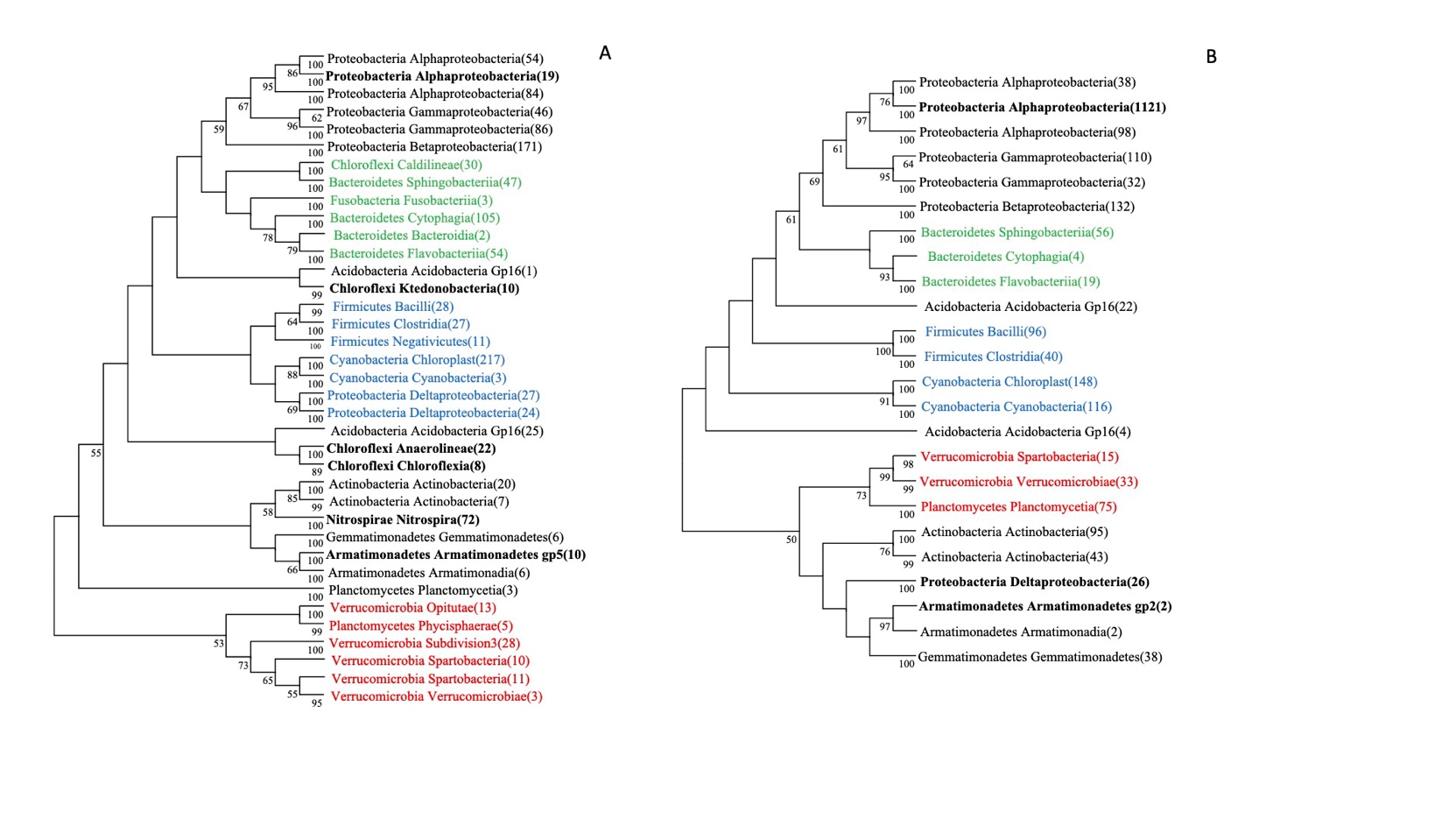


Fig. S2
